# Supplementary material for: Evolutionarily conserved susceptibility of the mitochondrial respiratory chain to SDHI pesticides and its consequence on the impact of SDHIs on human cultured cells
Source: PLoS One. 2019 Nov 7;14(11):e0224132. doi: 10.1371/journal.pone.0224132 (PMC6837341; doi:10.1371/journal.pone.0224132)
Supplement: S1 Table — (PDF) [file pone.0224132.s001.pdf]

Supplemental Table 1. **Permissive** *versus* **nonpermissive culture media for mitochondrial respiratory chain-deficient human cells.**

	Permissive medium GlucoMax	Nonpermissive medium MitoMax
Dulbecco modified Eagle's minimal essential medium (DMEM)	+	+
4.5 g/l glucose	+	-
4 mM glutamine	+	+
200 µM uridine	+	-
2 mM pyruvate	+	-
100 U/ml penicillin	+	+
100 U/ml streptomycin	+	+
10% fetal calf serum	+	+
