# Supplementary figures and images for: Evolutionarily conserved susceptibility of the mitochondrial respiratory chain to SDHI pesticides and its consequence on the impact of SDHIs on human cultured cells
Source: PLoS One. 2019 Nov 7;14(11):e0224132. doi: 10.1371/journal.pone.0224132 (PMC6837341; doi:10.1371/journal.pone.0224132)

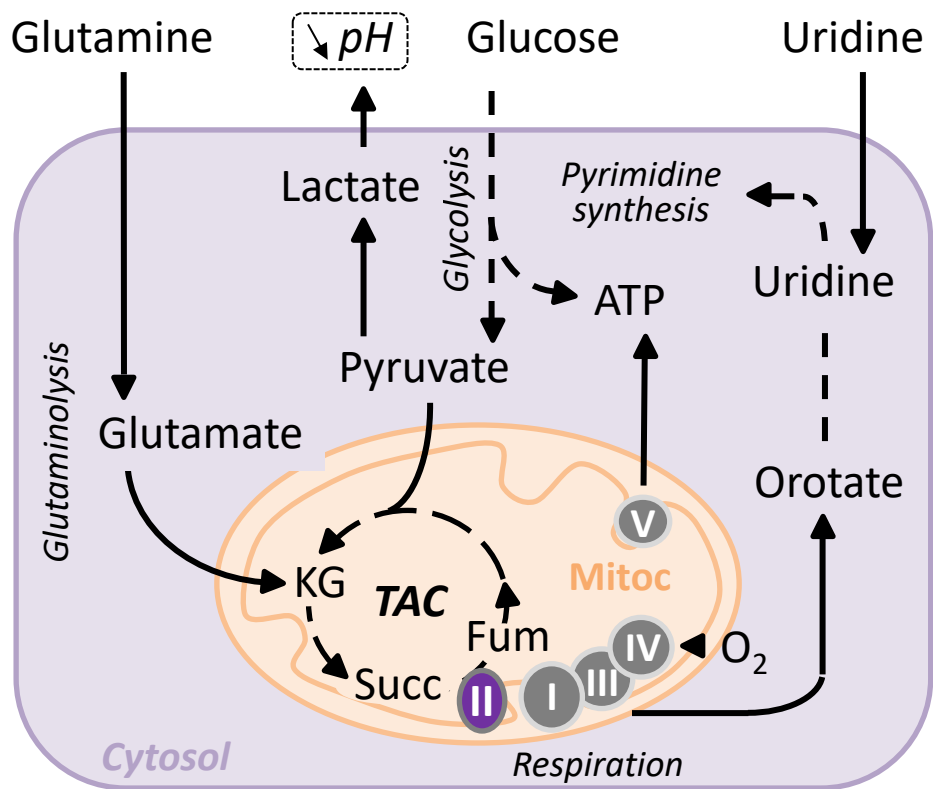

Suppl. Figure 1 B  nit et al

Supplement: S1 Fig — Fum, fumarate; KG, α-ketoglutarate; Succ, succinate; TAC, tricarboxylic acid cycle; I-V, the various complexes of the respiratory chain (II, complex II or SDH). (PDF) [file pone.0224132.s002.pdf]

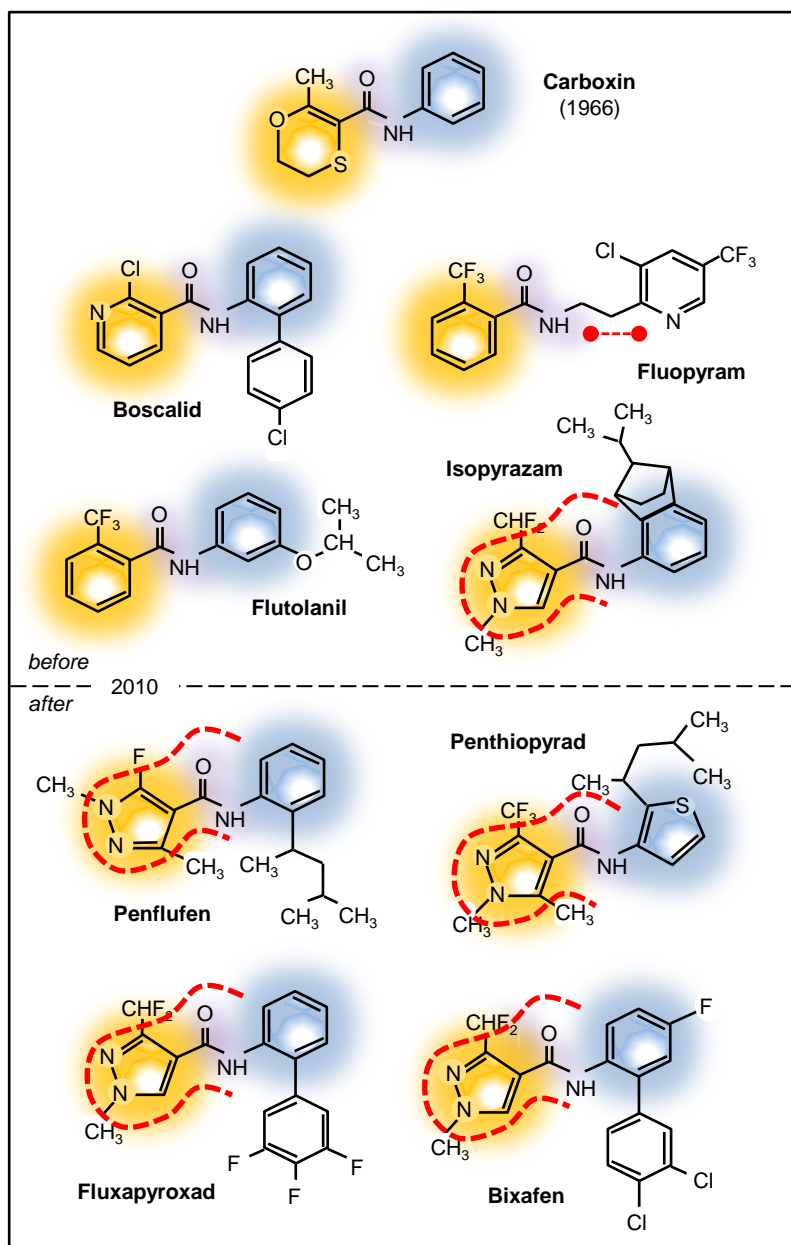

Supplement: S2 Fig — The yellow and blue parts for each molecule are reminiscent of the predecessor carboxin structure (top). The red dotted lines underline the methyl-pyrazol moiety present in the different SDHIs of the recent generation that also inhibit respiratory chain complex III. Boscalid (ADI: 0.04 mg/kg/d) was introduced in 2003 in the USA by BASF, fluopyram (ADI: 0.012 mg/kg/d) in 2010 in the USA by Bayer, flutolanil (0.09 mg/kg/d) in 1981 in the USA by Nichino America, penflufen (ADI: 0.04 mg/kg/d) in 2012 in the USA by Bayer, isopyrazam (ADI: 0.03 mg/kg/d) in 2010 in England by Syngenta, penthiopyrad (ADI: 0.1 mg/kg/d) in 2011 in the USA by Dupont-Fontelis, fluxapyroxad (ADI: 0.02 mg/kg/d)in 2011 in France by BASF, and bixafen (ADI: 0.02 mg/kg/d) in 2011 in England by Bayer. ADI; Acceptable Daily Intake according to European regulations. (PDF) [file pone.0224132.s003.pdf]

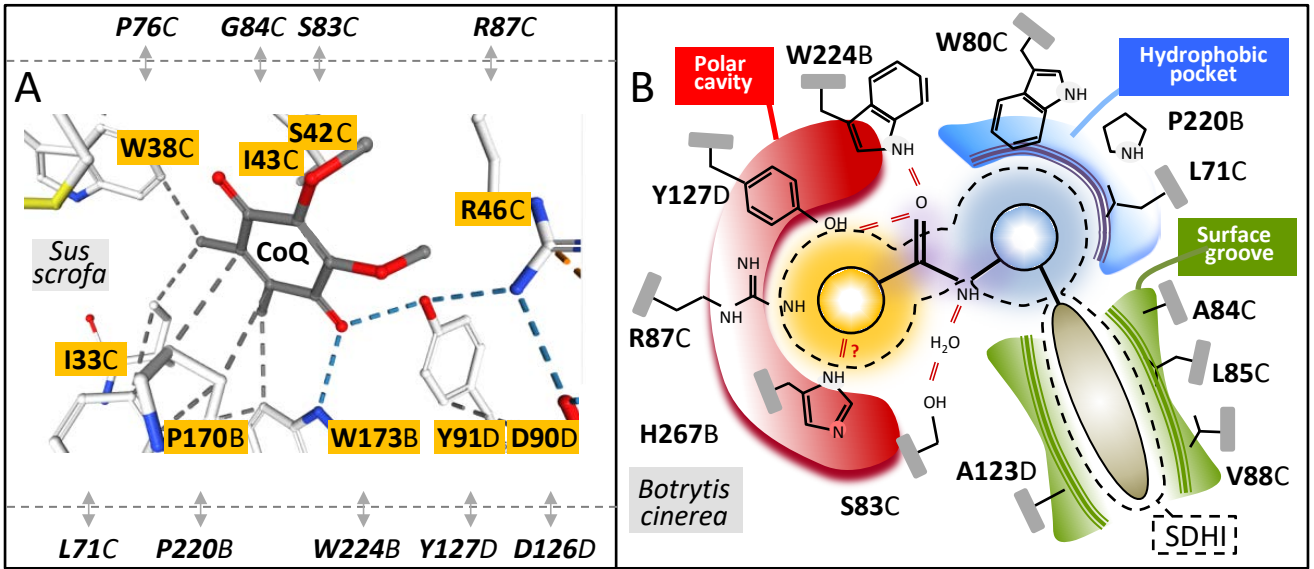

Bénit et al. Suppl. Fig. 3

Supplement: S3 Fig — A, Crystal structure of the quinone-binding site of the porcine (Sus scrofa) heart mitochondrial SDH (EC 1.3.5.1) drawn based on information from the Protein Data Bank archive. Amino acids are numbered corresponding to the porcine sequence using Roman characters, while the italic characters outside the figure correspond to the numbering of the Botrytis cinerea sequence. B, Schematic depiction of the ubiquinone-binding site of SDH featuring some of the amino acids that have been said to favor fungal resistance to SDHIs (Sierotzki and Scalliet 2013). Encircled by the dotted line, a simplified representation of SDHIs shows in yellow the part of the molecule accommodated by the polar cavity of the ubiquinone-binding site of SDH; the part accommodated by the hydrophobic pocket is shown in blue. (PDF) [file pone.0224132.s004.pdf]
