# Supplementary material for: Evolutionarily conserved susceptibility of the mitochondrial respiratory chain to SDHI pesticides and its consequence on the impact of SDHIs on human cultured cells
Source: PLoS One. 2019 Nov 7;14(11):e0224132. doi: 10.1371/journal.pone.0224132 (PMC6837341; doi:10.1371/journal.pone.0224132)
Supplement: S4 Fig — A, List of the 22 organisms selected for alignment with the references of each protein sequence as deposited in the NCBI database. B, C, D, For each subunit, the graphic representation illustrates the conservation (indicated in red) of the subunit among the 22 species selected, followed by the alignment showing the amino acids present at the positions (gray background/dark arrows) supposedly involved in the resistance of fungi to SDHIs or/and in the binding of coenzyme Q (S3 Fig) [29]. Alignment was performed using the COBALT multiple sequence alignment tool (https://www.ncbi.nlm.nih.gov/tools/cobalt/re_cobalt.cgi). (PDF) [file pone.0224132.s005.pdf]

A – 22 organisms selected for SDH B, C, D protein alignment.

Organism		SDHB	SDHC	SDHD
1. A mycorrhizal fungus having symbiotic relationships with plant roots	*Glomeromycota Rhizophagus irregularis*	EXX77477	PKY26074	EXX77069.1
2. A necrotrophic fungus affecting many plant species	*Botrytis cinerea*	ALK82288.1	ACT83441.1	ACT83439.1
3. A bacterium involved in mutualistic symbiotic relationship with legumes	*Rhizobium leguminosarum*	OBY07352.1	PUB61560.1	AIC29272.1
4. A fungus causing septoria tritici, necrotic blotches on wheat foliage	*Zymoseptoria tritici*	AER08706.1	AFV73771.1	AFV73750.1
5. A species of acroporid coral	*Acropora digitifera*	XP_0157542 11.1	XP_0157557 80.1	XP_0157557 81.1
6. The barrelclover, a small annual legume	*Medicago trunculata*	KEH43401.1	AES88117.1	AES71543.2
7. The vinegar fly, an irreplaceable genetic tool	*Drosophila melanogaster*	AAF57396	NP_001262 472.1	NP_651181. 1
8. The banana spider indigenous to both north and south America	*Nephila clavipes*	PRD39438.1	PRD21447.1	PRD34935.1
9. The small white, a small- to medium-sized butterfly species	*Pieris rapae*	XP_0221209 23.1	XP_0221277 16.1	XP_0221269 97.1
10. A free-living, transparent nematode, extensively used as a model organism.	*Caenorhabditis elegans*	BAA23717.1	CAA82572.1	CTQ86584.1
11. European honey bee the commonest of the 7–12 species of honey bee worldwide	*Apis mellifera*	XP_0262951 77.1	XP_0065649 13.1	XP_0011204 12.2
12. The African clawed frog found in the south-eastern portion of Sub-Saharan Africa	*Xenopus laevis*	NP_001080 247.1	XP_0180890 12.1	NP_001087 943.1
13. The zebrafish, a freshwater fish native to the Himalayan region, a favorite model organism	*Danio rerio*	AAI42800.1	AAH78306.1	AAH79507.1
14. The northern pike, a carnivorous fish found in the fresh waters from Northern hemisphere	*Esox lucius*	XP_0108797 58.1	ACO14487.1	XP_0108919 78.1
15. The common shrew, one of the most common mammals, throughout Northern Europe, but Ireland	*Sorex araneus*	XP_0046034 33.1	XP_0046139 49	XP_0046047 49.1
16. The European rabbit native to southwestern Europe and to northwest Africa	*Oryctolagus cuniculus*	ABD77138.1	XP_0082624 07.1	XP_0027084 82.1
17. The red junglefowl, the primary progenitor of the domestic chicken, was first domesticated at least 5000 years ago in Asia	*Gallus gallus*	NP_001074 344.2	XP_0249994 18.1	NP_001006 321.1
18. The domestic cat, the only domesticated species in the family Felidae	*Felis catus*	XP_0039895 72.1	XP_0039996 07.1	XP_0039924 03.1
19. The dingo, a dog that is native to Australia.	*Canis lupus dingo*	XP_0253031 86.1	XP_0252766 30.1	XP_0253213 30.1
20. The domestic sheep typically kept as livestock throughout the majority of the inhabited world	*Ovis aries*	XP_0120216 66.1	XP_0120234 95.1	XP_0120169 40.1
21. The wild boar, also known as the wild swine or wild pig, native to much of Eurasia, North Africa, and the Greater Sunda Islands	*Sus scrofa*	ABJ09403.1	XP_0031257 07.2	NP_001090 985.1
22. At the origin of the suicidal use of SDHIs, yet referred as *sapiens*	*Homo sapiens*	NP_002991. 2	CAG33383.1	CAG33213.1

B-SDHB

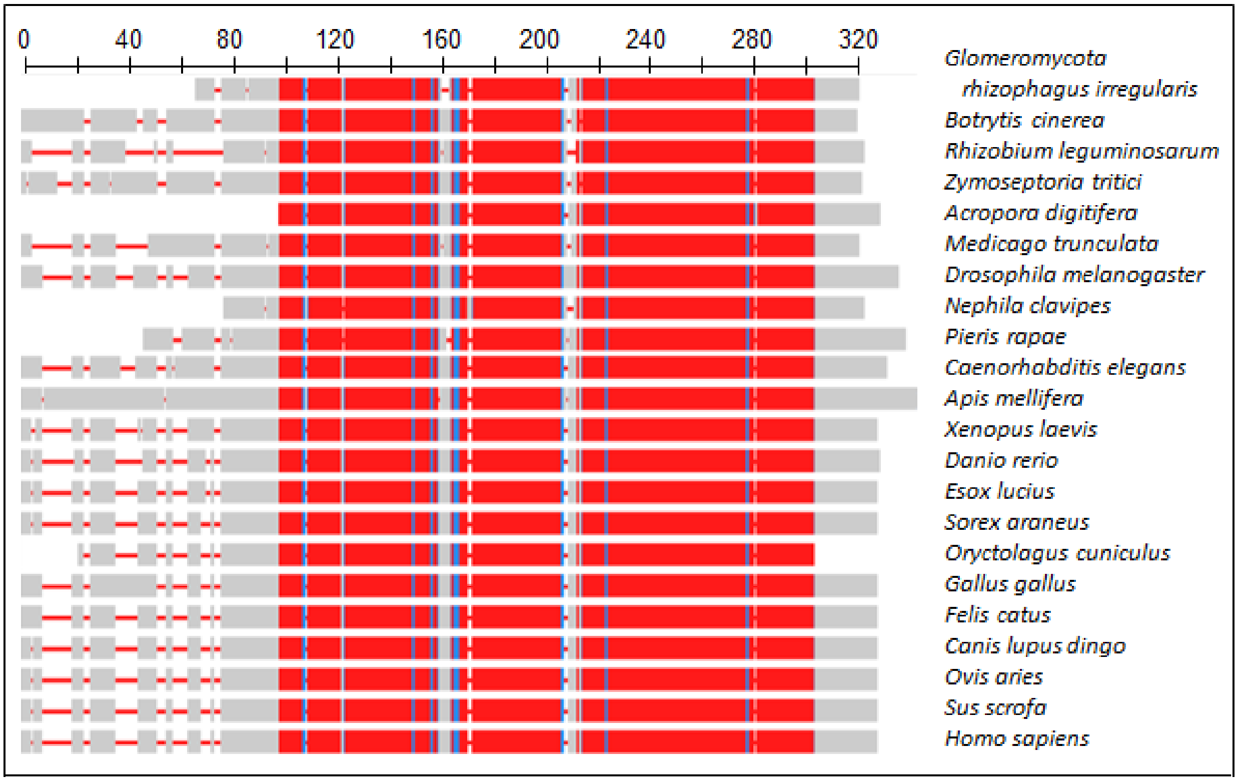


```
1    --------    ----------------   -------   --   ---MNLLsrA---TSRTIKINA-NTDPDKPSTKP
1    MAALrTGA[12]AFRT---QMRTMASVD[8]--VSPSR   PV[6]STVKEPAadS---ESLIKTFNIYRWNPDEPTSKP
1    MVEL----    ALPK---NSQMREGKV[4]------A   GA   --------------KNTREFRVYRWSPDD-GQNP
1    MA-LrLAT[ 6]AFRR---GMATTIE-H[9]EALSASR   PP[6]STVKEPQmdA---DAKTKTFHIYRWNPDQPTDKP
     --------    ----------------   -------   --   ----------------------------------
1    MAST----    MLKR---AIHRISSSP   ----TSR[4]RA[6]AQQASPKarD---TTVLKKFQIYRWNPDTP-SKP
1    MLATeARQ    ILSR---VGSLVARNQ[2]AISNGTA   QL   EQQAQPKeaQ---EPQIKKFEIYRWNPDNAGEKP
1    --------    ----------------   -------   --   --------------MQKLVFSIYRYNPDV-DQAP
1    --------    ----------------   --MNYFR[4]TA[2]YSTAKPKkqV---DPR-RIFKVYRFDGILTKGAP
1    MLAVrLVF    CTRR---SRRQRHSCC[3]ASTAAAA   EA[5]DDVAAKTkkT---GNRIKTFEIYRFNPEAPGAKP
1    MFNFnTIS[11]SLRRykcQNNRFRSHR[9]KPPSEED[3]TT[6]ECKTPPEerKidiKPRLQTIRVYRWNPEKPNVKP
1    MAAV--VF    SLRR---SGPVLRLSG   A-LQVSR   GA   QTAAAPAsqA---AARIKKFAIYRWDPDKPGDKP
1    MAAV-CFS    -LSR---CCSAVHRPA   --VTAVR   FA   QTAAAPA--A---QPRIKKFQIYRWDPDTVGDKP
1    MSVI-CFS    SLGR---CTAMAYRSS   AGIVAVR   YA   QTAAATA--T---QPKIKKFQVYRWDPDTVGDKP
1    MAAV-AAV    SLRR---RFAGAAVGG   ACXQACR   GT   QTAAA----A---APRIKKFAIYRWDPDKTGDKP
1    --------    --RR---RFPATALGG   VCLQACR   EA   QTAAA----T---APRIKKFAIYRWDPDKAGDKP
1    MAAAvVGV    SLRR---GVPARFLRA[9]AVQGICR   GA   QTAAA----A---TSRIKKFSIYRWDPDKPGDKP
1    MAAAaVGV    SLRR---RFPATALGG   ACLQACR   GA   QTVAA----T---APRIKKFAIYRWDPDKTGDKP
1    MAAV-VGV    SLKR---RFPAAALGG   ACLQACR   GA   QTAAA----T---APRIKKFAIYRWDPDKTGDKP
1    MAAV-VAL    SLRR---RFPAAALGG   ARLQACR   GA   QTAAA----A---APRIKKFAIYRWDPDKTGDKP
1    MAAV-VAV    SLKR---WFPATTLGG   ACLQACR   GA   QTAAA----T---APRIKKFAIYRWDPDKTGDKP
1    MAAV-VAL    SLRR---RLPATTLGG   ACLQASR   GA   QTAAA----T---APRIKKFAIYRWDPDKAGDKP
```

```
28   RLQQYKVDLNT-CGPMVLDALIKIKNEQDATLTFRRSCREGICGSCAMNINGTNTLACICKID---RLDTKIY--PLPHM
83   RMQSYTLDLNK-TGPMMLDALIRIKNEVDPTLTFRRSCREGICGSCAMNIDGVNTLACLCRIPRDAKHETKIY--PLPHT
44   SIDTFYIDVDD-CGPMVLDGLLYIKNKIDPTLTLRRSCREGICGSCAMNIDGTNTLACTKGLD-DIKGAVKIY--PLPHL
81   RMQSYTLDLNK-TGPMMLDALIRIKNEVDPTLTFRRSCREGICGSCAMNIDGVNTLACLCRIPTDTAKETRIY--PLPHT
1    -MQTYEVDLNR-CGPMVLDALIKIKNEIDPTLTFRRSCREGICGSCAMNIGGTNTLACISRINPDENKKTKIY--PLPHM
63   ELKEYEINLKE-CGPMVLDALIKIKNDIDPSLTFRRSCREGICGSCAMNIDGCNGLACLTKIP-DAGMDSTIT--PLPHM
64   YMQTYEVDLRE-CGPMVLDALIKIKNEMDPTLTFRRSCREGICGSCAMNIGGTNTLACISKIDINTSKSLKVY--PLPHM
20   RMQDYTLDNPEgRDMMVLDALLKLK-EQDPTLSFRRSCREGVCGSDGLNMNGKNGLACITPLSAVGLKGGKIVlrPLPGL
44   EMKNYDLDVST-CGKMVLDALIKIK-DMDPTLAFRRSCREGICGSCAVNLQGKNCLACITEIPPD--KKITIY--PIPHM
70   TVQKFDVDLDQ-CGTMILDALIKIKNEVDPTLTFRRSCREGICGSCAMNIGGQNTLACICKIDSDTSKSTKIY--PLPHM
97   YMQQFSVDLNKcTGTMVLDVLALIKAEYDPTLSYRKSCREGICGCCAMNINGVNNLACITKA-LESSKPIVIY--PLPHS
59   RMQTYEVDLNT-CGPMVLDALIKIKNEVDPTLTFRRSCREGICGSCAMNINGGNTLACTVRIDTNLSKVSKIY--PLPHM
56   RMQTYEIDLNT-CGPMVLDALIKIKNEMDSTLTFRRSCREGICGSCAMNINGGNTLACLNKIDTNTSKVTKIY--PLPHM
59   RMQTFEIDLNT-CGPMILDALIKIKNEMDGTLTFRRSCREGICGSCAMNINGGNTLACLNKIDTNTSKVTKIY--PLPHM
57   RMQTYEVDLNK-CGPMVLDALIKIKNEIDSTLTFRRSCREGICGSCAMNINGGNTLACTRRIDTNLNKVSKIY--PLPHM
48   RMQTYEVDLNK-CGPMVLDALIKIKNEIDSTLTFRRSCREGICGSCAMNINGGNTLACTRRIDTNLDKVSKIY--PLPHM
67   RMQTYEVDLNK-CGPMVLDALIKIKNELDSTLTFRRSCREGICGSCAMNIAGGNTLACTKKIDPDLSKTTKIY--PLPHM
58   RMQTYEIDLNK-CGPMVLDALIKIKNEIDSTLTFRRSCREGICGSCAMNINGGNTLACTRRIDTNLSKVSKIY--PLPHM
57   HMQTYEIDLNK-CGPMVLDALIKIKNEIDSTLTFRRSCREGICGSCAMNINGGNTLACTRRIDTNLSKVSKIY--PLPHM
57   HMQTYEIDLNN-CGPMVLDALIKIKNEIDSTLTFRRSCREGICGSCAMNINGGNTLACTRRIDTNLSKVSKIY--PLPHM
57   HMQTYEIDLNN-CGPMVLDALIKIKNEIDSTLTFRRSCREGICGSCAMNINGGNTLACTRRIDTNLDKVSKIY--PLPHM
57   HMQTYEVDLNK-CGPMVLDALIKIKNEVDSTLTFRRSCREGICGSCAMNINGGNTLACTRRIDTNLNKVSKIY--PLPHM
```

```
102  YVIKDLVPDLTHFYKQYKSIEPYLKKKNVP--KDGDRENLQSIADRKKLDGLYECILCACCSTSCPSYWWNSDEYLGPAV
159  YVVKDIVPDLTQFYKQYKSIKPYLQHTDPAp--EGK-EYLQSKEDRKKLDGLYECILCACCSTSCPSYWWNSEEYLGPAI
120  PVVKDLVPDLTNFYAQHRSIEPWLKTVSPAp----AKEWKQSHEDRQKLDGLYECILCACCSTSCPSYWWNGDRYLGPAV
158  YVVKDLVPDMTQFYKQYKSIKPYLQRDTAPp--DGK-ENRQSVADRKKLDGLYECILCACCSTSCPSYWWNSEEYLGPAV
77   YVVKDLVPDMTNFYEQYRSIEPYLKKKTEV--EYGKEQYLQSIEDRKKLDGLYECILCACCSTSCPSYWWNGDKYLGPAV
139  FVIKDLVVDMTNFYNQYKSIEPWLKRKSPAe--EDGKEIKQSKKDRAKLDGMYECILCACCSTSCPSYWWNPESYLGPAA
141  YVVRDLVPDMNNFYEQYRNIQPWLQRKNEAgeKKGKAQYLQSVEDRSKLDGLYECILCACCSTSCPSYWWNAEKYLGPAV
99   PVIRDLVVDMSQFYTQYEKVKPYLINDSKLp---PAGEFLQSPEERAKLDGLYECILCACCSTSCPSFWWNPDKFIGPAG
118  YVIRDLVVDMTHFFNGYDSIRPYLIRHDRQk-KLGSHQYAQSIEDNNKLVGLYECVLCACCSTSCPSYWWNGRRFLGPAT
147  FVVKDLVPDMNLFYAQYASIQPWIQKKTPL--TLGEKQMHQSVAERDRLDGLYECILCACCSTSCPSYWWNADKYLGPAV
174  YVIRDLVTDLEQYLKQYKNIEPFLKRTGEDn-YVGLRQILQSPRDRDKLNGLYECILCGCCTFACPPYWWLGDKFLGPST
136  YVVKDLVPDLSNFYAQYKSIEPYLKKKDKS--QQGKEQYLQSIEDRDKLDGLYECILCACCSTSCPSYWWNADKYLGPAV
133  YVVKDLVPDMSNFYAQYKSIEPYLKKKDES--QQGKQQYLQSVEDRQKLDGLYECILCACCSTSCPSYWWNADKYLGPAV
136  YVVKDLVPDMSNFYAQYKSIEPYLKKKDET--NEGKEQYHQTVEDRQKLDGLYECILCACCSTSCPSYWWNGDKYLGPAV
134  YVIKDLVPDLNNFYAQYKSIEPYLKKKDES--QEGKKQYLQSIEDREKLDGLYECILCACCSTSCPSYWWNGDKYLGPAV
125  YVIKDLVPDLSNFYAQYKSIEPYLKKKDES--QGGKQQYLQSIEEREKLDGLYECILCACCSTSCPSYWWNGDKYLGPAV
144  YVVKDLVPDLSNFYAQYKSIEPYLKKKDES--KQGKEQYLQSIEDRQKLDGLYECILCACCSTSCPSYWWNGDKYLGPAV
135  YVIKDLVPDLSNFYAQYKSIEPYLKKKDES--QEGKQQYLQSIEDREKLDGLYECILCACCSTSCPSYWWNGDKYLGPAV
134  YVIKDLVPDLSNFYAQYKSIEPYLKKKDES--QEGKQQYLQSIEDREKLDGLYECILCACCSTSCPSYWWNGDKYLGPAV
134  YVIKDLVPDLSNFYAQYKSIEPYLKKKDES--QGGKEQYLQSIEDREKLDGLYECILCACCSTSCPSYWWNGDKYLGPAV
134  YVIKDLVPDLSNFYAQYKSIEPYLKKKDES--QEGKQQYLQSIEEREKLDGLYECILCACCSTSCPSYWWNGDKYLGPAV
134  YVIKDLVPDLSNFYAQYKSIEPYLKKKDES--QEGKQQYLQSIEEREKLDGLYECILCACCSTSCPSYWWNGDKYLGPAV
```

Bénit et al. Suppl. Fig 4 B (cont.)

```
180   LLQAYRWMIDSRDDYSSERREAL-QNPFSVYRCHTIMNCANTCPKGLNPGHAIAMIKRDMALD-------
235   LLQSYRWLADSRDQKKEERKAAL-DNSMSLYRCHTILNCSRTCPKGLNPGLAIAEIKKEMAF--------
196   LLQAYRWLIDSRDEATGERLDNL-EDPFRLYRCHTIMNCAQTCPKGLNPAKAIAEIKKMMVERRV-----
235   LLQSYRWINDSRDEKTAQRKDAL-NNSMSLYRCHTILNCSRTCPKGLNPALAIAEIKKSMAFTG------
155   LMQAYRWMIDSRDDYTEERLEKLdSDPFKVYRCHTIMNCTKTCPKGLNPGKAIGEIKKMLASYQSKKAAT[ 1]
217   LLHANRWISDSRDEYTKERLEAI-NDEFKLYRCHTILNCARACPKGLNPGKQIAHIKSLQPKA-------
221   LMQAYRWIIDSRDENSAERLNKL-KDPFSVYRCHTIMNCTRTCPKGLNPGRAIAEIKKLLSGLASKPAPK[ 8]
176   LLHAYRFLADSRDTATEQRLNDL-DDAFSVFRCHGIMNCVNVCPKGLNPTKAIGQIKSMLLNRAL-----
197   LLHAYRWIIDSRDQDSEARLYDL-KDDFAVFRCHTIMNCTLACPKGLAPHDYIARLKHLISRISKKPQPS[11]
225   LMQAYRWVIDSRDDYATERLHRM-HDSFSAFKCHTIMNCTKTCPKHLNPAKAIGEIKSLLTGFTSKPAAE[ 4]
253   LLQAYRWIIDSRDMGHKERLTKL-RDYYSVYRCHTIFNCTKTCPKGLNPGKAVAQIKRLLAGLAKKDRPD[20]
214   LMQAYRWMIDSRDDFTEERLSKL-QDPFSLYRCHTIMNCTRTCPKGLNPGKAIAEIKKMMAMYKERAVSA
211   LMQAYRWMIDSRDDFTEDRLSKL-QDPFSLYRCHTIMNCTRTCPKGLNPGKAIAEIKKMMVTYKQKDAVA[ 1]
214   LMQAYRWMIDSRDEFTEERLSQL-QDPFSLYRCHTIMNCTRTCPKGLNPGLAIAEIKKMMAMYKEKRAAA
212   LMQAYRWMIDSRDDFTEERLAKL-QDPFSLYRCHTIMNCTKTCPKGLNPGKAIAEIKKMMATYKEKKASA
203   LMQAYRWMIDSRDDFTEERLAKL-QDPFSLYRCHTIMNCTRTCPKG------------------------
222   LMQAYRWMIDSRDDYTEERLAQL-QDPFSLYRCHTIMNCTRTCPKGLNPGKAIAEIKKMMATYKEKAAAA
213   LMQAYRWMIDSRDDFTEERLAKL-QDPFSLYRCHTIMNCTRTCPKGLNPGKAIAEIKKMMATYKGKKASV
212   LMQAYRWMIDSRDDFTEERLAKL-QDPFSLYRCHTIMNCTRTCPKGLNPGKAIAEIKKMMATYKGKKASV
212   LMQAYRWMIDSRDDFTEERLAKL-QDPFSLYRCHTIMNCTRTCPKGLNPGKAIAEIKKMMATYKEKQASA
212   LMQAYRWMIDSRDDFTEERLAKL-QDPFSLYRCHTIMNCTGTCPKGLNPGKAIAEIKKMMATYKEKKASA
212   LMQAYRWMIDSRDDFTEERLAKL-QDPFSLYRCHTIMNCTRTCPKGLNPGKAIAEIKKMMATYKEKKASV
```

C – SDH C

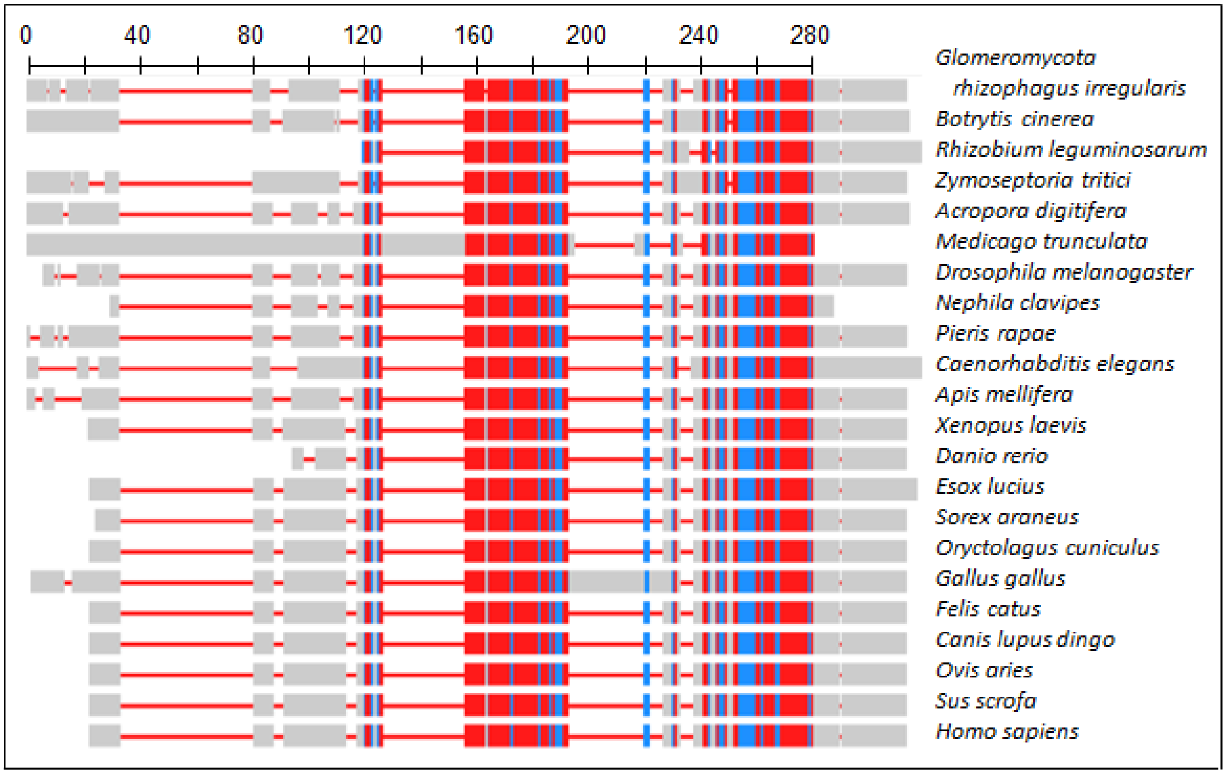


```
1   [19]-RTSMTRNGVF    SILKRN-    --TRQDTTSATSQGTSQEEI--    -LVSQR-AK    RPLSPFM-IYQPQ
1   [22]SQLAMRKLAAP    AAIGAS-    MQTRPVATQKLTPKDSYN-I--    -LVEQR-KL    RPVAPHLTIYQPQ
1       -----------    -------    ----------------------    --MANVTNN    RPLSPHLQIYKPI
1   [21]------RFATP    AAIALGN[4]QQRRQVTAAAVSESHARNEI--    -LAKQR-LN    RPVAPHLAIYKPQ
1   [20]SSVLTRRCLRP    SCLLMRS    ---TTITTSSLV----RSEE--[1]FWSKNSRLK    RPMSPHLTVYKPQ
1   [22]TFSSIPRSTGS[48]NSLRYQA[4]TNVLAGARYGTQVSGTKSPMLL[4]LMARNIERG[30]RPLSPHLPVYQPQ
1   [ 9]RSPA-LRQGLQ    MAAASRP    ---VSMKVVSVA--ETQKDE--[1]FFEKNERLG    RELSPHLTIYQPQ
1       --------MSA    FLILSSH    ---LPVTTSAAR----VATE--[1]FFEKNERLK    RPLSPHLSIYKLQ
1   [15]GSVAKLRPALR    TAQYASS    ---ATVPKISFKPYEPPKDE--[1]FDVKNERLN    RPMSPHLTIYKPQ
1   [ 8]----LCRLGAR    SSISRS-    -----FGTSIVTKSEAKTPIQK[4]YLLKQRSKN    RPIAPHLTVYQPQ
1   [ 9]SRRCIDPCTFR    NFYTCSS    ---RNIAVSKPLFKETTICE--[1]HDEKNLRLK    RPLSPHLTIYQIQ
1       MAALFLRHAGR    QCLRTQL    SPLLGARHVVPMGTTAQQEMER    FWNKNNRLS    RPLSPHMTVYKWS
1       -----------    -------    ---MLFQ----WGLHLMRKWTS    FGQKNTRLN    RPMSPHMTIYKWS
1       MALFLRSLTRQ    GTCLSRS    QFGVLYRHAVPMGTTAKEEMNK    FWAKNNKLN    RPMSPHVTVYKWS
1       --VLSCRHVGR    HCLRAHV    GPRLCIQNAAAMGTTAKEEMER    FWRKNMGSN    RPMSPHITIYSWS
1       MAALLLRHVGR    HCLRAHL    SPQLCIRNAVPLGTTAKEEMER    FWNKNTSSN    RPLSPHITIYSWS
1   [19]VQRDELRCVAR    RCLLARL    SPGPSVHHVVPMATTAKEEMAR    FWEKNTKSS    RPLSPHISIYKWS
1       MAALLLRQVGH    HCLRAHL    SPQRCIRNAVPLGTTAKEEMER    FWNKNAGLN    RPLSPHITIYSWS
1       MAALLSRHVGR    HCLRAHL    SPQLCIRNAVPLGTTAKEEMER    FWNKNTDLN    RPLSPHITIYSWS
1       MAALLLRHVGR    HCLRAHL    SPQLCIRNAVPLGTTAKEEMER    FWKKNTSSN    RPLSPHISIYSWS
1       MAALLLRHVGR    HCLRAHL    SPQLCIRNAVPLGTTAKEEMER    FWNKNLGSN    RPLSPHITIYRWS
1       MAVLLLRHVGR    HCLRAHF    SPQLCIRNAVPLGTTAKEEMER    FWNKNIGSN    RPLSPHITIYSWS
```

```
73   LTWYMSFAHRATGAGLAAGLYGTI    AA    YAFGGP    DSDTLVAAVSTL--PPTLKLAGKFCISYAFTYHTFNGI
78   IPWIMSGLNRITGCILSGGFYVFG    AA    YLASPL[5]DTASMVAAFGAW--PLAAKFLAKFTLAMPFTYHSFNGL
21   PTMVMSIVHRITGGALYVGTVLVA    WW    LIAAAS[3]---SYD--WANWVLGSLLGKLVLLGYTWALLHHMLGGF
78   ITWYLSALNRVTGVAASGAFYAFG    LL    YLAAPS[5]ESAALAASFGAW--PVLLQVLTKTILALPVTFHSLNGV
75   LTSMLSITHRGTGIAMTAVTVSFA    LA    ALALPG    NFEHYLGLVKALEIPAWIIFSGKTVLAWPLSYHSFNGI
171  LSSTLSICNRIAGAFLAAVTLLFY[ 5]VG    ---LVG[1]---TFDPFYQFVFYSSKLHLLAIEISALAMSYHLYSAI
65   LTSMLSICHRGTGLALGVGVWGLG    LG    ALISSH    DISHYVTMVEGLQLSGATLTALKFIIAYPAGYHTANGI
47   ITTLLSVTHRATGIVLSGGLYAFS    IG    MLALPG    HFPYYFEYFQTLHIAAPIIFSLKFALAWTTLYHSANGI
74   LTSLLSVTHRAAGMLLSGYITALG    VG    ALVLPN    DVAHYVTMIEGLNLSPATIFLAKACLAAPLGYHFANGI
65   LTWMLSGFHRISGCVMAGTLLVGG    IG    FAVLP-[1]DFTAFVDFIRSWNLPCAVTAVFKYIIAFPIIFHTLNGI
68   LTAFLSITHRTTGMILSSYAMLFG    IG    TLLIPG    GIPCLIEIISELGLSAPVLFVGKTLLALPATYHTFNGL
63   LPMAMSIIHRGTGVAMSAGVSMFG    LA    ALVLPG    DFASYLELVKSLSLGPALIYSAKFALAFPLAYHTWNGV
38   VPMAMSISHRGTGIALSSGISAFA    LA    ALVLPE    SYPYYLDLIHSLTFGPQFLAFSKFALAFPVVYHTYNGI
63   VPMMMSISHRGTGLGLSAGVSAFA    LL    ALVLPG    SYPYYLDLIHSQSFGPALIGLAKFGIAFPLSYHTFNGI
61   LPMMMSICHRGTGVALSAGVSLFG    LS    ALLVPG    NFESHLELLRSLSLGPALIHSAKFALAFPLMYHTWNGV
63   LPMAMSICHRGTGVALSAGVSLFG    LS    ALLLPG    NFESYLELVKSLSLGPALVHTAKFALVFPLMYHTWNGV
82   LPMAMSITHRGTGVALSLGVCTSG[27]VS[5]ALLLPE    QFPHYVAVVKSLSLSPALIYSAKFALVFPLSYHTWNGI
63   LPMAMSICHRGTGMALSAGVSLFG    LS    ALLVPG    NFESHLELVKSLCLGPSLIYTAKFALVFPLMYHTWNGI
63   LPMAMSICHRGTGVALSAGVSLFG    LS    ALLVPG    NFESHLELVKSLCLGPSLIYTAKFALVFPLMYHTWNGI
63   LPMAMSICHRGTGIALSAGVSLFG    LS    ALLVPG    SFESHLEFVKSLCLGPALIHTAKFALVFPLMYHTWNGI
63   LPMAMSICHRGTGIALSAGVSLFG    LS    ALLLPG    NFESHLELVKSLCLGPTLIYTAKFGIVFPLMYHTWNGI
63   LPMAMSICHRGTGIALSAGVSLFG    MS    ALLLPG    NFESYLELVKSLCLGPALIHTAKFALVFPLMYHTWNGI
```

```
141  RHLIWDTGKALTIK-GVYGTGYAVLILSTLSSIVLSVI
151  RHLAWDMGKTFKNA-TVVKTGWTVVGLSVGSALALVAF[ 1]
89   RHFMWDLGYGFGKE-FSTKLAIANIIGSLCLTVLVWVI[ 6]
151  RHLVWDTASMITNK-QVQTTGWTVVGLSVASALGLAFL
145  RHLAWDLGYGFDIG-ILYKSGWFVFFGSILAAASLAYF[ 1]
241  RHLFI---------------------------------
135  RHLLWDTGRFLKIK-EVYSTGYAMVATSFVLSAILALL
117  RHLPTSNCKLFL--------------------------
144  RHLYWDTAKGLTIK-EVYSTGYAMLAGAVAISLFLAAL
135  RFLGFDLAKGVNNVgQIYKSGYLVSGLSAILALAIVFN[10]
138  RHLAWDLGMFLTIK-EVYSTGYAVIALSAISAIALAAL
133  RHLIWDLGKGFKIP-QLYQSGITVLALTLITAVGLAAM
108  RHLAWDAGKGFKIP-EVYRSGYVVLGLTVLTSIGLAAM
133  RHLCWDSGKGFKIP-EVYGSGYTVIVLTVLTTIAAVAY[ 4]
131  RHLMWDLGKGLKIP-QLYQSGVAVLVLTVLTSAGLAAL
133  RHLMWDLGKGLKIP-QLYQSGVAVLVLTVLSSVGLAAM
184  RHLVWDMGKGFKLS-QVEQSGVVVLILTLLSSAAIAAM
133  RHLMWDLGKGLKIP-QLYQSGVAVLVLTVLSSVGLAAM
133  RHLIWDLGKGLKIP-QLYQSGVAVLVLTVLSSVGLAAM
133  RHLMWDLGKGLTIS-QLHQSGVAVLVLTVLSSVGLAAM
133  RHLIWDLGKGLTIP-QLTQSGVVVLILTVLSSVGLAAM
133  RHLMWDLGKGLKIP-QLYQSGVVVLVLTVLSSIGLAAI
```

Bénit et al. Suppl. Fig. C (end)

D - SDH D

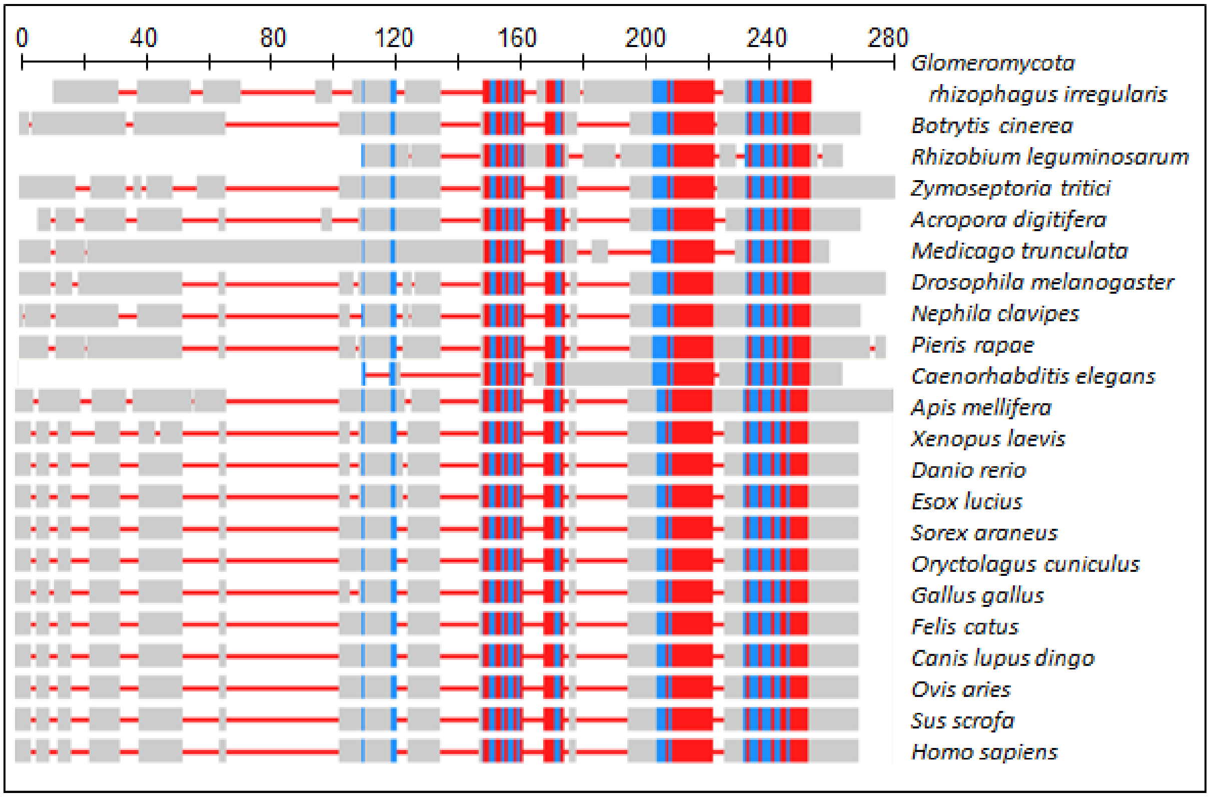


```
1   ---------------mYSTS[6]AAGSSSLLRT   SMTRNGVFSILKRN[ 1]RQ[17]----SQRAKRPLSPFMIYQP
1   --MASF-i-KPSVirqTCLA[6]STKIPSSFPA[3]PAGRSTFVRDALPG[12]QS    ILPPLPQSIDGTSNDAAVP
1   --------------------   ----------   --------------   --    -------MDMRTPLGKVRGL
1   --MASTAl-RPAAlrqLLTA[2]-TKRASTLPA[3]L--RTQFTQRS---[ 7]RP    ILPPLPQVIRGGVNDPAPVK
1   --MAAVMcaRLGF--rISRP   ------LARS[3]PIFAPSSISSCPRV   QS    VFF---KKTIHSSANHNTEL
1   --MQSVAl-KLGK--rLSNS[5]RISPNFIAHA[6]PSPPSPPISAAENA[12]AP[37]SLEGTGKAAIKVPPHTVAGY
1   msLSLLL--RGAV---RCNA[4]KSARITPLKS[6]NVQRKAVVQPLAVA   KI    VAPV--VREISVSAPRMA-S
1   --MIAVF--NVAR--nNCRG[6]QSKAQSLSNG   NLVAQKEIS-LLKQ   KP    TALFSTSHRLCAAAAAHD--
1   --MAFSMffRAPA----CTG[5]QIMRLATKPV[6]SSIMPLQTS-FRKS   DT    TPILNAVRSFRTSPALLHGE
1   --------------------   ----------   --------------   --    -------M--------LSG-
1   miLKSVAs--PSIfhkVRQL[3]-SKTTLFANS[4]QFTRKSSNFAIFKQ[11]QL    LKFPTNTIILNTEITRKTST
1   --MVTVL--RLSS---LCRA   --NRASAFKS   LLIRP--VPCLSQD   LH    TVQ---TSQIHTSQNHHA--
1   --MAALV--RISS---LCHR   GVSPLLFRPS   SLIRPLAVQQKDHD   CS    YLI---SARIHATPSNYAGS
1   --MAAIV--RISS---LCHR   GVKPLFHRSS   LLLRPLGVQQKDQD   RP    YQL---TARIHGSPSLYSGS
1   --MAVLW--RLGV---LCGA   QGGRALLLRS   QVVRPACVSAFLQD   PS    SVRYCGVQNIHLSPSRHA--
1   --MATLG--RLSV---LCGA   QGSRAVFLRS   LVVRPTHVAAFLQD   RP    SPGWCGVQHIHLSPSRHS--
1   --MAALV--LLRA--gLARP   RGVPTALLRG   TLLRHSAVLTAAAD   RS    APA---RQSHGGAPQGHG--
1   --MANLW--RLGV---LCRA   QGGRALFLRI   PVVRPAYVSAFLQD   QP    APGQCRTQHIHLSPNHHA--
1   --MASLW--RLSV---LCGA   QGGRALFLRI   PVVRPAHVSAFLQD   QP    APGWCRTQHIHLSPNHYS--
1   --MATLW--RLSV---LCGV   REGRALLLRT   PVVRPALVSAFLQG   RP    AQGWCGTQHIHLSPSHRS--
1   --MATLW--RLSV---LCGA   RGGGALVLRT   SVVRPAHVSAFLQD   RH    TPGWCGVQHIHLSPSHQA--
1   --MAVLW--RLSA---VCGA   LGGRALLLRT   PVVRPAHISAFLQD   RP    IPEWCGVQHIHLSPSHHS--
```

Bénit et al. Suppl. Fig D

```
72   q---LTWYMSFA     -HRATGAGLAAGLY[3]AAYAFGgpDS[16]AGKFCISYAFTYHTFNGIRHLIWDTGK[1]LTI
67   kpSPSHGSYHWT     FERLIAVGLVPLTV   APFVSGslNP    ATDALLCAAILIRSHIGFESCITDYFP[3]VPK
14   g-SAKEGTSHFW     RQRLTAVSNIPLIL[7]AYAGAPy---[13]VVMGLMVISGVIHMKLGMQVIIEDYVH[2]FGK
70   epSPSHGSYHWT     MERLVSAALIPLTI   VPFAAGslNP    VLDGTFIGMIIIHSYIGFQSAITDYFP[3]VPK
57   eiAPSQPSSHWK     FERYVSVAMLTLIP   TGIIYP--SA    AVDWALAVVVPIHNHWGVGQVLTDYIH   GST
122  atAATAGEVDIK[13]TKREQLLKVTAAVP   LLLIYPnaYS[ 7]---------IFWHINAGIEEILADYVH   ---
69   --AGSSHTLLWT     VERIVSAGLLAVIP   AAFIAP--SQ    VLDALMAISVVIHTHWGVEAMVVDYMR[4]GNV
64   ----HSHSRIWT     AERFLSAALIGILP   AAVAFP--NP    VLDYVLALSMTVHVHWGIEAIVVDYVR[4]GAV
71   k--AHDHSKLWV     IEKVTSAALVPLIP   ICLMIP--NK    LFDSVLAILITAHSFWGLEAIAVDYVR[4]GPI
5    ------------     FHRISGCVMAGTLL[4]GFAVLPfdFT[17]VFKYIIAFPIIFHTLNGIRFLGFDLAK[2]NNV
82   t--ASDHVRMWI     LEKIASAALPVIIP   VALTME--NV    ICDGLMSLLIVIHMHWGLEAIITDYAR[4]GPL
51   --ASKAASLHWT     SERALSVALLGLLP   AAYLYP--GA    AVDYSLAAALTLHGHWGLGQVVTDYVH   GDA
57   --GSKAATMHWT     GERILSIALLSLAP   VAYFCP--SP    AVDYSLAAALTLHGHWGLGQVVTDYVH   GDA
57   --GSKAASLHWT     GERVVSVMLLALGP   AAYYIP--GP    AIDYSLAAAITLHSHWGIGQVLTDYIH   GES
58   --GSKAASLHWT     SERVVSVLLLGLLP   AAYLSP--CS    AIDYSLAATLSLHSHWGLGQVVTDYVP   GAT
58   --GSKAASLHWT     GERVVSVLLLGLLP   AAYLNP--CS    AMDYSLAAVLSLHSHWGLGQVVTDYVH   GDA
56   --SSKAASLHWT     SERAVSALLLGLLP   AAYLYP--GP    AVDYSLAAALTLHGHWGLGQVITDYVH   GDT
58   --GSKASSLHWT     GERVVSVLLLGLIP   AAYLNP--SS    AMDYSLAAALTLHSHWGLGQVVTDYVH   GDA
58   --GSKAASLHWT     GERVVSVLLLGLIP   AAYLNP--GS    AMDYSLAAALTLHSHWGLGQVVTDYVR   GDG
58   --GSKAASLHWT     GERVVSVLLLGLIP   AAYLNP--CS    AMDYSLAATLTLHSHWGIGQVVTDYVH   GDA
58   --SSKAASLHWT     GERVVSVLLLGLLP   AAYLNP--CS    AMDYSLAAALTLHGHWGIGQVVTDYVR   GDA
58   --GSKAASLHWT     SERVVSVLLLGLLP   AAYLNP--CS    AMDYSLAAALTLHGHWGLGQVVTDYVH   GDA
```

```
154  KGVYGTGYAVLILSTLSSIVLSVI----------------
136  TKAFLWWGLRGATVLVGVGLYEFETNDVGVTEGIKRIWRA
98   ---IALLMLNTFFAIVIAGLCLFAIL--KIAFVG------
139  TRKLADWANVAAVFLVGWGWYEFETNDIGLTAGIARVWTA[15]
121  MPKLAKGLWLAVSILQFIGLCYFNYTDVGICKAVCMIWHM
196  HEMTREFVLISLRLFLIIAIKDVFLNFVFV----------
135  LPKVAHIALIIISVATLGGLFYFIQNDVGLANGIKRFWAI[ 8]
128  LSKAAVATVYALSVLTLGGLFYFNYTDVGLSQAIRMLWKL
137  IPKIAIALVYLISIATLGGLFYLISNDVGMSNAIRQLWGV[ 6]
82   GQIYKSGYLVSGLSAILALAIVFNSCQNKSNKTA------
148  LPKLLHLSLIFLSAITLCGLFLLINNGPGVSKAIKEAWAI[14]
113  KIKLANTSLFALSALTFAGLCYFNYHDVGICKAVAMLWSL
119  KIKMANAGLFVLSTVTFAGLCYFNYHDVGICKAVALLWSK
119  KIKVANACLFLLSTVTFAGLCYFNHNDVGICKAVALLWSK
120  TQKAAKAGVLALSALTFAGLCYFNYHDVGICKAVAMLWKL
120  PQKAAKAGLLALSALTFAGLCYFNYHDVGICKAVAMLWKL
118  PIKVANTGLYVLSAITFTGLCYFNYYDVGICKAVAMLWSI
120  LQKTAKAGVLALSALTFAGLCYFNYHDVGICKAVAMLWKL
120  LQRAVKAGVLALSALTFAGLCYFNYHDVGICKAVAILWKL
120  VQKAAKTGLLVLSAFTFAGLCYFNYHDVGICKAVAMLWKL
120  LQKAAKAGLLALSAFTFAGLCYFNYHDVGICKAVAMLWKL
120  LQKAAKAGLLALSALTFAGLCYFNYHDVGICKAVAMLWKL
```

Bénit et al. Suppl. Fig. D (end)
