# Supplementary material for: Evolutionarily conserved susceptibility of the mitochondrial respiratory chain to SDHI pesticides and its consequence on the impact of SDHIs on human cultured cells
Source: PLoS One. 2019 Nov 7;14(11):e0224132. doi: 10.1371/journal.pone.0224132 (PMC6837341; doi:10.1371/journal.pone.0224132)
Supplement: Attachment — Submitted filename: Reviewer.docx [file pone.0224132.s006.docx]

Responses to reviewer’s comments

**We first like to thank the reviewer for his/her positive appreciation of our manuscript.**

**We carefully considered all the points and try to change the manuscript according the suggestions.**

*1 -Material and Methods: - Human cultured fibroblasts and HEK cells -
The two types of media are described at the beginning of this paragraph but are not named immediately. The two names GlucoMax and MitoMax are shown only at the end of the paragraph. This is a bit confusing.*

**We have now modified the manuscript as to refer to the two culture media at the head of the section.**

*Results section: - Effects of SDHIs on the mitochondrial respiratory chain -
It would help to report the SDHIs concentrations used in agriculture as a comparison for the IC50 results.*

**We have on one side attempted to calculate what could be these concentrations and for this introduced a new paragraph in the last discussion. The difficulty lies in the fact that if it is relatively easy to know what is distributed in the field, the actual result for the exposed organisms in the field is obviously dependent on so many factors that such calculation is much more erratic. In addition, for the sake of comparison, we have now intraduced when possible the ADI for each SDHI (eg legend of S2 Figure).**

*- SDHIs and the viability of cultured human cells. Half life of SDHIs? The experiments last for 12/14 days without the medium getting changed. The differences seen between the compounds could be due to differences in their respective half lives.*

**The half-lives of these molecule exceed several months in the nature, an observation that rules out that such parameter could represent a major issue in our study**

Fig. 4, B: only the graph for bixafen in permissive medium is reported. Could you show the fluxapyroxad graph in permissive medium as well?
**We have modified the figure to insert a new set of data showing the lack of effect of Fluxapyroxad in permissive medium**

Minor corrections:
L206/207: this sentence is a bit confusing. Try rephrasing.

**Done**

L253: the IC50 is reported only for bixafen and not for isopyrazam. Both of them are discussed in this sentence, therefore I would include both IC50 values.

**The IC50 was already reported for both molecules in Figure 2**

L256: the IC50 reported for bixafen on CIII is 15.3 µM, the table says 15.5 µM

**Corrected**

L290: add "C" inside the brackets (Fig 3A, B and C) not (Fig 3A, B)

**Done**

L321 to 324: this sentence is not very clear. Try rephrasing.

**Hopefully better, at least we tried**

L329: the "D" needs to be bold.

**Done**
